# Measuring the level of compulsory hospitalisation in mental health care: The performance of different measures across areas and over time

**DOI:** 10.1002/mpr.1881

**Published:** 2021-05-25

**Authors:** Tore Hofstad, Jorun Rugkåsa, Solveig O. Ose, Olav Nyttingnes, Tonje L. Husum

**Affiliations:** ^1^ Centre for Medical Ethics University of Oslo Oslo Norway; ^2^ Health Services Research Unit Akershus University Hospital Lørenskog Norway; ^3^ Centre for Care Research University of South‐Eastern Norway Porsgrunn Norway; ^4^ SINTEF Health Services Research Trondheim Norway; ^5^ R&D Department, Division of Mental Health Akershus University Hospital Lørenskog Norway

**Keywords:** compulsory hospitalisation, geographic variation, measurement, small area analysis

## Abstract

**Objective:**

A variety of measures are used for reporting levels of compulsory psychiatric hospitalisation. This complicates comparisons between studies and makes it hard to establish the extent of geographic variation. We aimed to investigate how measures based on events, individuals and duration portray geographical variation differently and perform over time, how they correlate and how well they predict future ranked levels of compulsory hospitalisation.

**Methods:**

Small‐area analysis, correlation analysis and linear regressions of data from a Norwegian health registry containing whole population data from 2014 to 2018.

**Results:**

The average compulsory hospitalisation rate per 100,000 inhabitant was 5.6 times higher in the highest area, compared to the lowest, while the difference for the compulsory inpatient rate was 3.2. Population rates based on inpatients correlate strongly with rates of compulsory hospitalisations (*r* = 0.88) and duration (*r* = 0.78). 68%–81% of ranked compulsory hospitalisation rates could be explained by each area's rank the previous year.

**Conclusion:**

There are stable differences in service delivery between catchment areas in Norway. In future research, multiple measures of the level of compulsory hospitalisation should ideally be included when investigating geographical variation. It is important that researchers describe accurately the measure upon which their results are based.

## 1 INTRODUCTION

Health service delivery is based on ethical principles of voluntariness and respect for autonomy (Etchells et al., 1996). Compulsory care, such as compulsory psychiatric hospitalisation represents a breach of individual autonomy and should therefore be used as a last resort and in the patient's best interest, and this is usually reflected in legislation that regulates such practice (Saya et al., 2019). Nonetheless, considerable concerns about current practice have been raised (Molodynski et al., 2016). This is, among other things, due to variation in the extent to which compulsory hospitalisation is used. A number of studies report practice variation when comparing the level of compulsory hospitalisation between countries (Hansson et al., 1999; Riecher‐Rössler & Rössler, 1993; Salize & Dressing, 2004; Sheridan Rains et al., 2019; Zinkler & Priebe, 2002) and within areas regulated by the same legislation (Bindman et al., 2002; Engberg, 1991; Keown et al., 2016; Kjellin, 1997). For instance, the highest admission rates among mental health departments in the Veneto region in Italy 2000–2007 were 14 times higher than the lowest rates (Donisi et al., 2016), while in France in 2012, the rate of compulsory hospitalised patients in the highest 10th percentile of psychiatric sectors' catchment areas was 10 times higher than the 90th percentile (Gandré et al., 2018). Such large differences indicate that some areas might use more compulsion than necessary. If that is the case, it would constitute *unwanted variation* which is described as ‘variation that cannot be explained on the basis of illness, medical evidence or patient preference’ (Wennberg, 2010, p. 4). Geographical variation is commonly observed in most health service delivery and might reflect differences in local practice, culture or service dimensioning. Given the implications of compulsory interventions for personal autonomy, it is particularly important to detect potential geographical variation in such practice, as a precursor to identify ways of improving the organisation and delivery of services in the best interest of patients.

In order to establish the extent of geographical variation in compulsory hospitalisations, both within and between jurisdictions, reliable measures are required. Trustworthy measures are also needed to track compulsion use over time, which would be necessary to monitor progress towards reduced compulsion levels, following political ambitions. However, there is a long lamented lack of agreement on standardisation of measuring and reporting compulsory mental health care, both in national statistics and the research literature (de Stefano & Ducci, 2008; Høyer, 2008; Riecher‐Rössler & Rössler, 1993; Sheridan Rains et al., 2019; Zinkler & Priebe, 2002). This heterogeneity complicates comparisons, as different studies often employ different measures of the level of compulsory hospitalisation and also because the descriptions of how measures are operationalised is not always clear. Studies that compare measures from various sources or areas are therefore conventionally accompanied by a note of caution.

Based on our reading of the research literature we have categorised the most frequently used measures according to three concepts, which are also fundamental to general epidemiology (Rothman et al., 2008): events, individuals and duration. *Event* measures are most common and are usually based on counts of compulsory admissions (Riecher‐Rössler & Rössler, 1993; Zinkler & Priebe, 2002). Some studies employ counts of *individuals* with minimum one compulsory hospitalisation (Gandré et al., 2018; Keown et al., 2016; Sheridan Rains et al., 2019; Weich et al., 2017). A number of studies include measures based on *duration*, normally the number of days between admission and discharge (Aguglia et al., 2016; Girolamo et al., 2008; Wierdsma & Mulder, 2009), although it varies whether studies include days of voluntary stay during an admission (Iversen et al., 2009; Kelly et al., 2018).

In order to make comparisons between countries, regions or institutions, raw numbers are often recalculated to population‐based rates per person year. Quotas or shares of compulsory hospitalisations/inpatients of the total number of hospitalisations/inpatients is sometimes used. While quotas can be useful when the population‐at‐risk is unknown, they are less suitable for comparison between areas, since they rely heavily on the total admission frequency, which is dependent on the available resources and coverage of mental health care in each country (Høyer, 2008; Riecher‐Rössler & Rössler, 1993). Quotas are therefore not part of this study.

While a more nuanced picture of the level of compulsory hospitalisation is likely to emerge through the inclusion of multiple measures, data availability might restrict options. It is therefore important to understand whether and how the selection of measures impacts inferences drawn about the level of compulsory hospitalisation, to limit biases due to methodology and choice of operationalisation.

Increasingly comprehensive, reliable and accessible hospital registries with individual level data hold potential for more valid estimates of variation in health care delivery, including variation in the level of compulsory hospitalisation. The Norwegian Patient Registry (NPR) ranks among the world's most complete and reliable health registries (Bakken et al., 2020) and enables us not only to describe the level of compulsory hospitalisation within selected samples, but the entire population. This also makes it possible to apply different measures, over an extended period of time, and to compare them across health care regions regulated by the same legislation. We have not found any study that has investigated how the commonly used measures appear and diverge when applied to a whole population, over time and how the resulting picture of variation might depend on the choice of measure. Therefore, this will be the focus of our study. Specifically, we aim to answer the following research questions:

1) How does the extent of geographical variation in the level of compulsory hospitalisation vary when captured by different measures and over time?
2) How do different measures applied to one population correlate?
3) How well can the different measures predict future ranked levels of compulsory hospitalisation in different areas?

## 2 METHODS

### 2.1 Setting

Our research questions will be addressed by calculating, and comparing and contrasting measures based on every episode of compulsory psychiatric hospitalisation in Norway in 2014–2018. In Norway, all use of psychiatric compulsion is managed by the specialist services. These are the responsibility of four Regional Health Authorities that are owned by the State. A total of 356 municipalities are nested within catchment areas of 65 Community Mental Health Centres (CMHC), which are part of the specialist service and constitute our main level of analysis. The annual populations of the catchment areas ranged from 6636 to 160,909 during the study period.

Compulsory hospitalisation is regulated by the 1999 Norwegian Mental Health Act. The main legal criterion is the presence of severe mental illness. In addition, the patient's condition must be likely to deteriorate without treatment, or the patient poses an immediate danger to themselves or others. Section 3.2 permits compulsory observation in hospital, which may last 10 days, and can be extended for another 10 days. Section 3.3 permits compulsory mental health care. Here, no time limit applies, but there are legal requirements to assess whether the criteria are met every 3 months. Section 3.4 prohibits the compulsory detention of patients admitted voluntary, except when there is immediate and serious danger to the patient or others. A legislation change in 2017 restricted compulsory care to patients without the capacity to consent to treatment, unless there is immediate danger. As intended, compulsion numbers were reduced initially, but figures rose again the following year (Bremnes & Skui, 2020).

### 2.2 Data inclusion

Data on all episodes of compulsory hospitalisation in the period 2014–2018 was obtained from NPR. The registry is person identifiable through unique patient numbers, which enabled us to follow individuals over time and across institutions. Only the *population at risk* of compulsory hospitalisation was included in the study, which was defined as all individuals between 18 and 65 years living in a Norwegian municipality during the study period. The age scope was primarily selected because services are organised differently for other age groups. Forensic admissions were not included, as this can be considered a distinct population, regulated through a different legal framework (Salize & Dressing, 2004).

Analyses were based on residency prior to each hospitalisation. This is likely to result in more homogeneous categories compared to analyses of where people were treated, since the numbers will not be impacted by service organisation (Wennberg, 2010). One percent of all patients relocated and were subsequently readmitted compulsory in a different area the same year. They were counted in both areas.

Population counts for municipalities, stratified by age and gender were collected from Statistics Norway (2020). Individuals without a Norwegian national identity number or missing values on residency were excluded (1% and <0.001% of persons compulsory hospitalised, respectively). The population of homeless people in Norway is small; 0.75 homeless per 1000 inhabitants (Dyb & Lid, 2017). Individuals in this category were included based on their last recorded residency.

Data completeness was in general good. The 11 catchment areas in Central Norway were excluded in 2014 due to incomplete reporting, but all areas were included in 2015–2018. In two cities, two CMHCs operate within the same urban district. Since we lacked sufficiently detailed information on residence for those living in these areas to determine to which CMHC they belonged, we combined the relevant two areas in both cities. The boundaries of some of the CMHC's catchment areas changed during the study period, so the number of catchment areas included in analyses varied (*N* = 60_2014_/69_2015–2016_/67_2017_/65_2018_/74_2014–2018_).

### 2.3 Variables

#### 2.3.1 Events, individuals and duration

*Compulsory hospitalisation* was defined by the registered legal status of each event, and included compulsory observation, compulsory mental health care and episodes where the patient was admitted voluntarily, but legal status was later converted to compulsion. If a patient was transferred between wards or institutions during the same episode of compulsion, this was counted as part of the same hospitalisation. The number of new compulsory hospitalisations was counted for each calendar year.

*Compulsory inpatients* were defined as individuals with at least one compulsory hospitalisation and this was counted for each calendar year.

Duration of compulsion excluded voluntary days during the admission, and was calculated in two ways: for the *Compulsory Length of Stay* (LoS) *rate*, the total number of days of compulsory hospitalisation was counted for each year. This included compulsory hospitalisations that commenced in earlier years, and hospitalisations that extended into the following year. This was done to ensure that compulsory hospitalisations that lasted more than 1 year were also included, providing a more valid estimate of prevalence. For *median compulsory LoS* and *average compulsory LoS,* number of days of compulsory hospitalisation was counted for each completed hospitalisation, after discharge, each year. This included days of compulsory hospitalisations that commenced in earlier years.

#### 2.3.2 Measures

The following measures of the level of compulsory hospitalisation were calculated for each CMHC catchment area for each year of the study.

1. Compulsory hospitalisation rate: number of compulsory admissions that year, divided by the population at risk, multiplied by 100,000.
2. Compulsory inpatient rate: number of compulsory admitted patients that year, divided by population at risk, multiplied by 100,000.
3. Compulsory LoS rate: annual number of days of compulsory hospitalisation, divided by the population at risk, multiplied by 100,000.
4. Median compulsory LoS: median number of days of compulsory hospitalisation.
5. Average compulsory LoS: average number of days of compulsory hospitalisation.

### 2.4 Statistical analysis

In order to answer the first research question of the extent of geographic variation when measured in different ways, four analyses were conducted to test how much each measure varied between areas. First, the extremal quotient (EQ) was calculated to quantify the magnitude of difference, by dividing the highest with the lowest rate. Second, while the EQ is a popular measure, it is impacted by the number of cases compared (Kazandjian et al., 1989). Therefore, to reduce the influence of outliers, the ratio of the 90th percentile to the 10th percentile (EQ_90/10_) of the distribution of each measure was also calculated (OECD, 2014). Third, the coefficient of variation (CV), which is the ratio of the standard deviation to the mean was calculated. The CV is useful for comparing the different measures of the level of compulsory hospitalisation, since the CV is scale invariant and shows the relative variability. Fourth, while both EQ and CV might be impacted by unstable rates due to small populations and rare occurrences, the systematic component of variation (SCV), which is specifically developed to investigate geographical variation, subtracts the random component from the variance (McPherson et al., 1982). For the hospitalisation rate and the LoS rate, the adjusted formula for SCV suggested by Cain and Diehr (1992) and Diehr et al. (1993) was employed, which accounts for the extra Poisson variation introduced by individuals with multiple admissions.

For all variation measures, higher values indicate higher levels of variation between areas. Univariate scatter plots were created for each measure to aid in the analysis. These included average scores for each CMHC area, grouped by health region.

In order to answer the second research question on the relationships between measures, Spearman's rank order correlation coefficient was calculated to investigate the pairwise relationships between the different measures of compulsory hospitalisation.

The third research question concerning the ability to predict future ranked levels of compulsory hospitalisation was answered by ranking each CMHC area for each year according to each measure of compulsion. Linear regression was performed using each year's rank as independent variable, and the subsequent year's ranking as dependent variable. This procedure was repeated for each compulsion measure, and *R* ^2^ was used as measure of explained variance. As a further test of stability, we performed the same analysis with ranks from 2014 as independent variable, as well as ranks based on the average values from 2014 to 2017, to predict ranks in 2018.

To reduce the impact of random fluctuation, the average over the 5‐year period for each area was used to answer research question one and two. In order to account for risk differences due to varying gender and age distribution, direct standardisation was performed for the rates, using the countrywide population as reference population (Ash et al., 2003). Expected cases were calculated based on six age categories (18–25, 26–33, 34–41, 42–49, 50–57, 58–65). Indirect standardisation using countrywide compulsory hospitalisation rates as reference was used for SCV. As robustness tests to avoid undue impact of unstable rates, all analyses were repeated without areas with less than 35,000 inhabitants, resulting in a reduction of catchment areas to 35–40.

All calculations were performed and figures generated in R‐version 3.6.2 (R Team, 2019), with the following packages: data.table 1.12.8 (Dowle et al., 2019), tidyverse 1.3.0 (Wickham et al., 2019), GGally 2.0.0 (Schloerke et al., 2020), lubridate 1.7.4 (Grolemund & Wickham, 2011) and fhidata: Structural Data for Norway (White, 2019).

### 2.5 Ethics

The Research Ethics Committee deemed the study to fall outside their remit as specified by the Norwegian Health Research Act (ref: 2018/795). After a detailed data protection impact assessment the study was approved by the Privacy Ombudsman at Akershus University Hospital (ref: 2018‐090). Prior to release from NPR, ID‐numbers were deidentified in accordance with relevant regulations.

## 3 RESULTS

During 2014–2018, 16,189 distinct individuals between 18 and 65 years were compulsory hospitalised 36,153 times in Norway, averaging 151 inpatients and 222 hospitalisations per 100,000 person per year. LoS for compulsory hospitalisations was 12 days (median) and 40 days (average). The total number of days of compulsory hospitalisation averaged 8514 per 100,000 person per year. A total of 423 individuals had their legal status changed after being admitted voluntarily, which equals 2.6% of all compulsory hospitalised patients.

### 3.1 Geographical variation by measure, health region and over time

Table 1 displays the average amount of geographical variation between CMHC areas during the study period. According to the EQ values, the difference between the CMHC areas with the highest and lowest values ranged from 3.2 to 8.4, depending on which measure of the level of compulsory hospitalisation was used. According to all four measures of variability, the inpatient rate showed least variability across areas. Most variation was seen among the measures based on duration.

**TABLE 1:** Variation in the level of compulsory hospitalisation among 74 CMHC areas in Norway 2014–2018

		EQ	EQ_90/10_	CV	SCV
Rates	Hospitalisations	5.6	2.4	35	10.9^a^
Per 100,000	Inpatients	3.2	2.0	26	5.1
Person per year	Los	8.0	2.9	40	16.5^a^
Median	Los	8.4	2.6	40	‐
Average	Los	4.9	2.9	36	‐

*Note:* Extremal quotient (EQ) = max/min. EQ_90/10_ = 90th decile/10th decile. Coefficient of variation (CV) = standard deviation/mean × 100. Systematic component of variation (SCV) × 100; reference for calculation in text.

Abbreviation: CMHC, Community Mental Health Centres.

^a^ SCV adjusted for multiple admissions.

**FIGURE 1:**
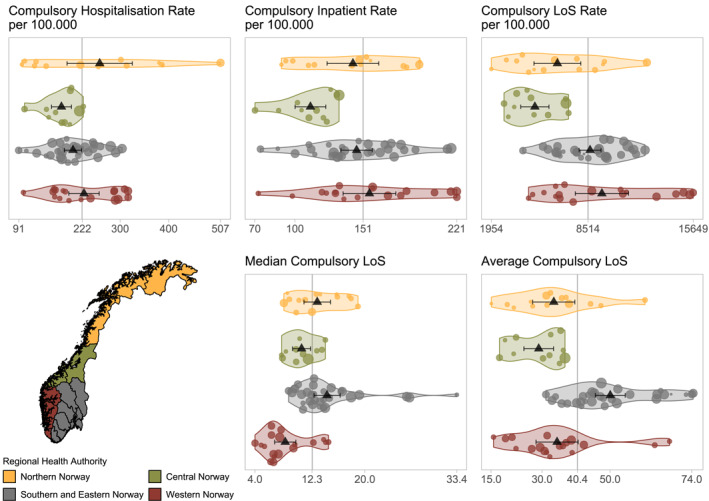
Scatter and violin plot of measures of the level of compulsory mental health care in Norway 2014–2018, grouped by Regional Health Authority. Scatter points represent the average value for each CMHC for the study period, with point size reflecting population count. Triangles show health region average with 95% confidence intervals. *X*‐axes include minimum, maximum and national average, which can also be seen in the vertical line. Map of Norway shows boundaries for Regional Health Authorities. CMHC, Community Mental Health Centre

Figure 1 shows the different measures applied to each CMHC area, grouped by health region. National average, minimum and maximum values are indicated on the *x*‐axis. Each compulsion measure painted a slightly different picture of the geographical variation in the level of compulsory hospitalisation. In Northern Norway outliers were seen for hospitalisation rates. Central Norway was lower than average on all measures, and also showed least variation within the health region. Median and average LoS were longer in Southern and Eastern Norway, and shorter than the country average for most CMHC areas in Western Norway.

**FIGURE 2:**
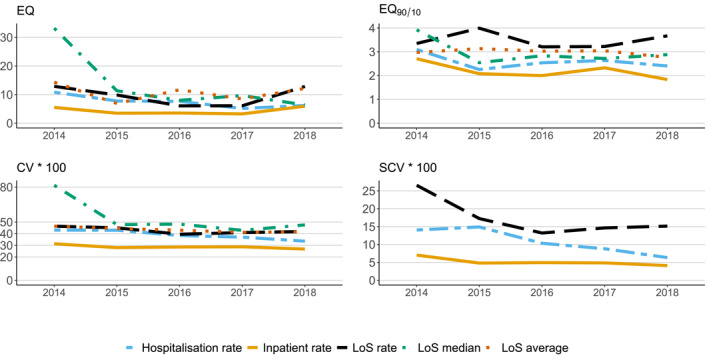
Variation in the average level of compulsory hospitalisation among 74 CMHC areas in Norway 2014–2018. Extremal quotient (EQ) = max/min. EQ_90/10_ = 90th decile/10th decile. Coefficient of variation = standard deviation/mean × 100. Systematic component of variation × 100. CMHC, Community Mental Health Centre

Figure 2 shows the variation in the different measures over the period 2014–2018. Inpatient rates consistently showed least variability for each year of the study compared with the other measures. Some reduction in geographic variation occurred during the study period, particularly the hospitalisation rate.

### 3.2 Correlation between measures

**FIGURE 3:**
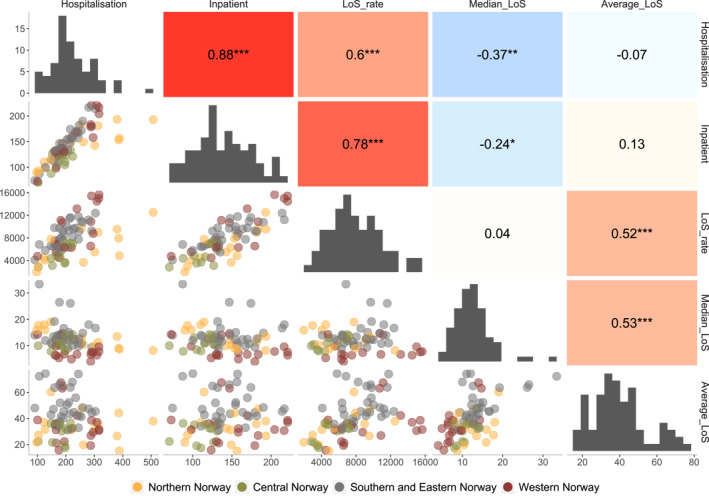
Correlation plot of measures of the level of compulsory mental health care in Norway 2014–2018. Upper half: heatmap with Spearman's rho. ***p < 0.001; **p < 0.01; *p < 0.05. Lower half: bivariate scatter plot. Diagonal: univariate distribution

Results from the correlation analyses are displayed in Figure 3. The strongest correlations were found between the hospitalisation rate and the inpatient rate (*r* = 0.88), and the inpatient rate and the LoS rate (*r* = 0.78). Negative correlations were seen between median LoS and the hospitalisation rate (*r* = −0.37), as well as between median LoS and the inpatient rate (*r* = −0.24). The upper left scatter plot shows that there is a ratio of roughly 2.5 between the highest hospitalisation rate (500) and the inpatient rate (200).

### 3.3 Predicting ranked levels of compulsory hospitalisation

**FIGURE 4:**
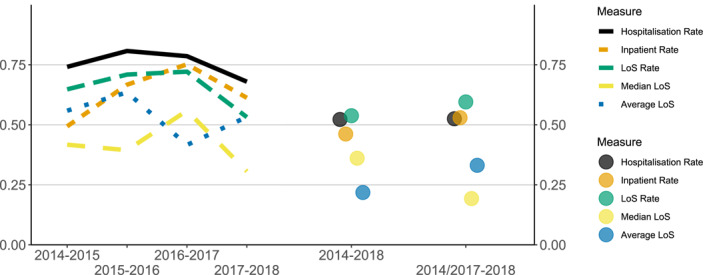
*R*^2^ from OLS. The left part of the figure shows the amount of explained variance in ranked levels of compulsory hospitalisation, as predicted from the previous year's levels. The right part shows explained variance for 2018 predicted by levels from 2014, and from the average levels during 2014–2017

Figure 4 displays *R* ^2^ from linear regressions of ranked measures. Hospitalisation rates were most useful for predicting ranks from year to year, explaining between 68% and 81% of the variation in the subsequent year's rank. 53%–60% of the variation in ranked rates in 2018 could be explained by the ranked average between 2014 and 2017.

### 3.4 Robustness test

The results of the robustness analyses without areas less than 35,000 can be seen in the Appendix Table A1. A reduction in geographical variation is observed, and predictions were improved.

## 4 DISCUSSION

To our knowledge, this is the first study to quantify geographical variation in the level of compulsory hospitalisation in mental health care for an entire population and over time, using different measures based on events, individuals and duration. The observed geographical variation was relatively stable across the years, and the variation in compulsory hospitalisation rates can be considered high compared to reference procedures (OECD, 2014). Furthermore, all duration measures showed considerable variation, suggesting substantial practice variation in the extent to which people remain compulsory hospitalised over time.

### 4.1 Inpatient rates vary less than hospitalisation rates

The number of compelled inpatients is necessarily less than or equal to the number of events in a period. Consequently, measures based on inpatient counts will by necessity never display more variation than measures based on hospitalisation counts when applied to the same dataset. How large the difference in variation will be is an empirical question which will differ between populations. In this study, population rates based on counts of inpatients displayed less than half the amount of geographical variation of rates based on hospitalisations and LoS according to the SCV, but the difference was reduced during the study period. The variation in inpatient rates can be categorised as medium‐to‐high variation compared to reference procedures (OECD, 2014). The scatter plot in the lower half of Figure 3 showed that in some areas the compulsory hospitalisation rate was roughly the same as the inpatient rate, which means that there were almost no compulsory readmissions each year in those areas. In contrast, the ratio of the hospitalisation rate to the patient rate was roughly 2.5 in the area with the highest hospitalisation rate. This means that every patient in that area on average had more than two compulsory hospitalisations each year. The consequences of whether rates based on events or individuals are used in research and official statistics is thus highly contingent on the practice of compulsory readmissions.

The extent of geographical variation was relatively stable over the years, particularly so for the compulsory inpatient rate, as displayed in Figure 2. Most geographical variation was seen in 2014 for most measures. Still, the extent of geographical variation regularly appears larger when considering numbers for each year separately, than the average for the whole period. This underlines the importance of caution when interpreting geographical variations based on numbers from a short time interval, since these might be impacted by fluctuation over time. By averaging over the 5‐year period, the more extreme differences due to random outliers were smoothed out. Substantial differences remained, however, indicating that there are stable differences in service delivery between different catchment areas of CMHCs.

The observed geographical variation pales in comparison with findings from catchment areas in France, where EQ_90/10_ for the inpatient rate was 10, and CV roughly 80% (Gandré et al., 2018). The EQ_90/10_ is impacted by the number of areas compared (Kazandjian et al., 1989), which was much larger in France (*N* = 514), and this can explain some of the discrepancy. While the CV is also a function of sample size, such large differences are not expected solely on the basis of the differing number of areas compared.

### 4.2 Inpatient rates correlate strongly with hospitalisation rates

Rates based on inpatients correlate strongly with rates based on hospitalisations in this study. In other words, areas where the level of compulsory hospitalisation is ranked higher, as measured by rates of hospitalisations, also tend to rank higher when measured by rates of inpatients. Despite the strong correlations, different health regions with higher or lower levels of compulsory hospitalisation than average stuck out, depending on measure. This implies that ranking areas using only one measure, might give different results based on the choice of measure.

Moreover, the correlation analysis showed that areas that tend to have shorter median duration of compulsory hospitalisations, also tend to have higher hospitalisation rates. An interpretation of this is that shorter compulsory LoS might not cause significant improvement in health, resulting in a need for further compulsory readmission. Another possible explanation is that some areas might require hospitalisation in order to administer involuntary depot medication, lowering the median and average LoS. On the other hand, the strong correlation between the LoS rate and both hospitalisation and patient rates indicate that more days are spent under compulsion in areas where more people are detained.

### 4.3 Compulsory hospitalisation rates predict future ranked levels of compulsory hospitalisation

The high amount of variance explained in ranked levels of compulsory hospitalisation, solely on the basis of last years ranking, suggest considerable stability over time. This has implications for efforts at reducing compulsion levels in high‐ranking areas. If random fluctuation dominates ranking, it would be difficult to identify areas that could benefit most from interventions to reduce compulsion levels. In this study however, areas that ranked higher one year were very likely to be ranked higher the subsequent year, particularly when ranked by the compulsory hospitalisation rate.

### 4.4 Ways of measuring: Events, individuals, duration

Researchers who are primarily concerned with the burden imposed on the hospitalised individuals in terms of infringed autonomy might emphasise different elements of the level of compulsory hospitalisation, than researchers who, for instance, set out to calculate costs associated with compulsory hospitalisations. The focus of each study might therefore impact the choice of measures.

Each compulsory hospitalisation will constitute additional breach of autonomy, so if the burden of compulsion experienced by patients in an area is under study, counts of events might provide better estimates than counts of individuals. Also, one individual who is subject to many events of compulsory hospitalisation might significantly impact the hospitalisation rate in an area, but not the inpatient rate. It is, therefore, an advantage of inpatient based rates that they are less prone to random fluctuation, which means that they will be more stable, even when data is only available for shorter time periods (Bowers, 2000).

A limitation with measures based on both events and individuals is that they do not distinguish between longer and shorter durations; one hospitalisation counts as one event regardless of length. The number of days under involuntary confinement thus provides an alternative take on the level of compulsory hospitalisation, and has been recommended in the compulsion literature (Høyer, 2008), but is not often reported. LoS is conventionally counted at discharge, which can be difficult to measure for individuals with very long involuntary hospitalisations. Those detained for more than a year might, in fact, not be included in annual statistics, although they in reality contribute 365 days to an area's total number of days of compulsory hospitalisation. The LoS rates in our study are therefore based on the actual number of days of involuntary hospitalisation for each year, regardless of which year the person was admitted or discharged. Arguably, this provides a better duration measure of the level of compulsory hospitalisation in an area. For average or median LoS however, including unfinished episodes would bias the estimate downwards.

### 4.5 National registers of compulsory admissions

This study was possible because of the existence of a national comprehensive population‐based registry (Bakken et al., 2020). Comprehensive health registries are required to analyse variation in compulsory hospitalisation at national levels, but are not available, or not of sufficient quality in many countries. As compulsory hospitalisation might compromise individual autonomy, a lack of high quality registers could constitute a democratic as well as a methodological problem.

### 4.6 The need for unambiguous terms

Høyer (2008) elaborated on difficulties in comparing rates of compulsory hospitalisation from different sources, since some studies only count involuntary admissions, while others include orders issued after admission; and the age range of the study population might differ and is often not reported. In a similar vein, based on the results from this study, we urge increased awareness of the importance of reporting accurately what measures are being used and how they are operationalised. At face value, terms that are commonly used in the research literature, such as *compulsory hospitalisation rate* and *involuntary admission rate* signify that they are based on *events*. But a closer reading shows that they are sometimes based on the number of patients who had at least one compulsory hospitalisation—in other words *individuals* (Gandré et al., 2018; Sheridan Rains et al., 2019; Weich et al., 2017). The extent to which rates based on hospitalisations or inpatients differ will depend on the compulsory readmission factor. As we have seen, the hospitalisation rate can be considerably larger, and vary substantially more, than the inpatient rate. Consequently, researchers should provide detailed and unambiguous descriptions of their measures, and what amounts to admission rates or patient rates should be named accordingly.

### 4.7 Further research

Further research is needed to establish the performance of measures of the level of compulsory hospitalisation in other populations. While our study focuses on inpatient compulsion it is clearly of interest to consider the extent and variation of outpatient commitment as well, which is another area of research that would benefit from adopting more standardised measures (Rugkåsa, 2016). It would be especially interesting to calculate continued duration of compulsion when inpatient stays result in discharge to outpatient commitment.

### 4.8 Strengths and limitations

A considerable strength of this paper is that we had access to complete information about all compulsory hospitalisations from the whole population in Norway over a 5‐year period. The data are routinely collected, which is simultaneously a strength and limitation with register data, since they are not primarily intended for research. Differences in the way events are registered can exist between areas. We rely on the evaluation of the NPR for the validity and completeness of the variables used. Furthermore, other risk factors that can be unevenly distributed, such as patient mix, were not accounted for. Consequently, it would not be right to label all the observed geographic variation as *unwarranted*.

## 5 CONCLUSION

This study showed that measures based on events, individuals and duration, paint different pictures of the extent and geographical variation of the level of compulsory hospitalisation. The geographical variation was high for hospitalisation rates and duration measures, and was stable across the years. Future studies describing the level of compulsory hospitalisation are likely to yield a richer and more accurate picture by including multiple measures. Since inpatient rates can be significantly lower than hospitalisation rates, and display less geographical variation, it is important that measures are accurately described to reflect how they are constructed and what they are based on.

## CONFLICT OF INTERESTS

The author declares that there is no conflict of interest.

## AUTHOR CONTRIBUTIONS

This study forms part of a larger research program for which Jorun Rugkåsa, in collaboration with Olav Nyttingnes and Tonje L. Husum obtained funding and accessed data. Tore Hofstad, Jorun Rugkåsa, Solveig O. Ose and Tonje L. Husum designed the present study. Tore Hofstad designed and performed the data analysis and wrote the first draft of the manuscript. All authors revised the manuscript in several rounds and approved the final version.

## Supporting information

Supplementary Material S1

Supplementary Material S2

Supplementary Material S3

Supplementary Material S4

## Data Availability

The data that support the findings of this study are available from The Norwegian Patient Registry. Restrictions apply to the availability of these data, which were used under license for this study.

## 1

**TABLE A1:** Variation in the level of compulsory hospitalisation among 40 CMHC areas in Norway 2014‐2018

		EQ	EQ_90/10_	CV	SCV
Rates	Hospitalisations	5.6	1.9	32	6.1^a^
Per 100,000	Inpatients	3.2	1.9	24	3.4
Person per year	Los	5.6	2.6	36	8.0^a^
Median	Los	6.6	2.4	39	‐
Average	Los	4.2	2.2	32	‐

*Note:* Population >35,000. Extremal quotient (EQ) = max/min. EQ_90/10_ = 90th decile/10th decile. Coefficient of variation (CV) = standard deviation/mean × 100. Systematic component of variation (SCV) × 100; reference for calculation in text.

Abbreviation: CMHC, Community Mental Health Centre.

^a^SCV adjusted for multiple admissions.

## References

[mpr1881-bib-0001] Aguglia, A., Moncalvo, M., Solia, F., & Maina, G. (2016). Involuntary admissions in Italy: The impact of seasonality. International Journal of Psychiatry in Clinical Practice, 20(4), 232–238. 10.1080/13651501.2016.1214736 27551753

[mpr1881-bib-0002] Ash, A. S., Shwartz, M., & Pekoz, E. A. (2003). Comparing outcomes across providers. Risk Adjustment for Measuring Health Care Outcomes, 3, 297–333.

[mpr1881-bib-0003] Bakken, I. J., Ariansen, A. M. S., Knudsen, G. P., Johansen, K. I., & Vollset, S. E. (2020). The Norwegian patient registry and the Norwegian registry for primary health care: Research potential of two nationwide health‐care registries. Scandinavian Journal of Public Health, 48(1), 49–55. 10.1177/1403494819859737 31288711

[mpr1881-bib-0004] Bindman, J., Tighe, J., Thornicroft, G., & Leese, M. (2002). Poverty, poor services, and compulsory psychiatric admission in England. Social Psychiatry and Psychiatric Epidemiology, 37(7), 341–345. 10.1007/s00127-002-0558-3 12111027

[mpr1881-bib-0005] Bowers, L. (2000). The expression and comparison of ward incident rates. Issues in Mental Health Nursing, 21(4), 365–374. 10.1080/016128400247988 11249355

[mpr1881-bib-0006] Bremnes, R., & Skui, H. (2020). Tvang i psykisk helsevern: Status etter lovendringene i 2017 (IS‐2888). Helsedirektoratet.

[mpr1881-bib-0007] Cain, K. C., & Diehr, P. (1992). Testing the null hypothesis in small area analysis. Health Services Research, 27(3), 267–294. 1500287 PMC1069879

[mpr1881-bib-0008] de Stefano, A., & Ducci, G. (2008). Involuntary admission and compulsory treatment in Europe: An overview. International Journal of Mental Health, 37(3), 10–21. 10.2753/IMH0020-7411370301

[mpr1881-bib-0009] Diehr, P., Cain, K., Ye, Z., & Abdul‐Salam, F. (1993). Small area variation analysis: Methods for comparing several diagnosis‐related groups. Medical Care, 31(5), YS45–YS53. 8492585

[mpr1881-bib-0010] Donisi, V., Tedeschi, F., Salazzari, D., & Amaddeo, F. (2016). Differences in the use of involuntary admission across the Veneto Region: Which role for individual and contextual variables? Epidemiology and Psychiatric Sciences, 25(1), 49–57. 10.1017/S2045796014000663 25487132 PMC6998663

[mpr1881-bib-0011] Dowle, M., Srinivasan, A., Gorecki, J., Chirico, M., Stetsenko, P., Short, T., Lianoglou, S., Antonyan, E., Bonsch, M., & Parsonage, H. (2019). Package ‘data.table’. Extension of ‘data.frame’ [Computer software].

[mpr1881-bib-0012] Dyb, E., & Lid, S. (2017). Homelessness in Norway 2016—A survey. NIBR. https://www.veiviseren.no/forstaa‐helheten/forskning‐og‐utredninger/rapport/bostedslose‐i‐norge‐2016‐‐‐en‐kartlegging

[mpr1881-bib-0013] Engberg, M. (1991). Involuntary commitment in Greenland, the Faroe Islands and Denmark. Acta Psychiatrica Scandinavica, 84(4), 353–356. 10.1111/j.1600-0447.1991.tb03159.x 1746287

[mpr1881-bib-0014] Etchells, E., Sharpe, G., Dykeman, M. J., Meslin, E. M., & Singer, P. A. (1996). Bioethics for clinicians: 4. Voluntariness. Canadian Medical Association Journal, 155(8), 1083–1086. 8873637 PMC1335358

[mpr1881-bib-0015] Gandré, C., Gervaix, J., Thillard, J., Macé, J.‐M., Roelandt, J.‐L., & Chevreul, K. (2018). Geographic variations in involuntary care and associations with the supply of health and social care: Results from a nationwide study. BMC Health Services Research, 18, 253. 10.1186/s12913-018-3064-3 29625567 PMC5889610

[mpr1881-bib-0016] Girolamo, G. de, Rucci, P., Gaddini, A., Picardi, A., & Santone, G. (2008). Compulsory admissions in Italy: Results of a national survey. International Journal of Mental Health, 37(4), 46–60. 10.2753/IMH0020-7411370404

[mpr1881-bib-0017] Grolemund, G., & Wickham, H. (2011). Dates and times made easy with lubridate. Journal of Statistical Software, 40(3), 1–25.

[mpr1881-bib-0018] Hansson, L., Muus, S., Saarento, O., Vinding, H. R., Göstas, G., Sandlund, M., Zandrén, T., & Öiesvold, T. (1999). The Nordic comparative study on sectorized psychiatry: Rates of compulsory care and use of compulsory admissions during a 1‐year follow‐up. Social Psychiatry and Psychiatric Epidemiology, 34(2), 99–104. 10.1007/s001270050118 10189816

[mpr1881-bib-0019] Høyer, G. (2008). Involuntary hospitalization in contemporary mental health care. Some (still) unanswered questions. Journal of Mental Health, 17(3), 281–292. 10.1080/09638230802156723

[mpr1881-bib-0020] Iversen, K. I., Høyer, G., & Sexton, H. C. (2009). Rates for civil commitment to psychiatric hospitals in Norway. Are registry data accurate? Nordic Journal of Psychiatry, 63(4), 301–307. 10.1080/08039480902730607 19199121

[mpr1881-bib-0021] Kazandjian, V. A., Durance, P. W., & Schork, M. A. (1989). The extremal quotient in small‐area variation analysis. Health Services Research, 24(5), 665–684. 2584039 PMC1065591

[mpr1881-bib-0022] Kelly, B. D., Curley, A., & Duffy, R. M. (2018). Involuntary psychiatric admission based on risk rather than need for treatment: Report from the Dublin Involuntary Admission Study (DIAS). Irish Medical Journal, 111(4), 736. 30488681

[mpr1881-bib-0023] Keown, P., McBride, O., Twigg, L., Crepaz‐Keay, D., Cyhlarova, E., Parsons, H., Scott, J., Bhui, K., & Weich, S. (2016). Rates of voluntary and compulsory psychiatric in‐patient treatment in England: An ecological study investigating associations with deprivation and demographics. British Journal of Psychiatry, 209(2), 157–161. 10.1192/bjp.bp.115.171009 27284079

[mpr1881-bib-0024] Kjellin, L. (1997). Compulsory psychiatric care in Sweden 1979–1993. Prevalence of committed patients, discharge rates and area variation. Social Psychiatry and Psychiatric Epidemiology, 32(2), 90–96. 10.1007/BF00788926 9050350

[mpr1881-bib-0025] McPherson, K., Wennberg, J. E., Hovind, O. B., & Clifford, P. (1982). Small‐area variations in the use of common surgical procedures: An international comparison of New England, England, and Norway. New England Journal of Medicine, 307(21), 1310–1314. 10.1056/NEJM198211183072104 7133068

[mpr1881-bib-0026] Molodynski, A., Khazaal, Y., & Callard, F. (2016). Coercion in mental healthcare: Time for a change in direction. BJPsych International, 13(1), 1–3. 10.1192/S2056474000000854 29093879 PMC5618885

[mpr1881-bib-0027] OECD . (2014). Geographic variations in health care: What do we know and what can Be done to improve health system performance? http://www.oecd.ilibrary.org/social‐issues‐migration‐health/geographic‐variations‐in‐health‐care_9789264216594‐en

[mpr1881-bib-0028] R Team . (2019). R: A language and environment for statistical computing version 3.0. 2. Vienna, Austria: R Foundation for Statistical Computing.

[mpr1881-bib-0029] Riecher‐Rössler, A., & Rössler, W. (1993). Compulsory admission of psychiatric patients—An international comparison. Acta Psychiatrica Scandinavica, 87(4), 231–236. 10.1111/j.1600-0447.1993.tb03363.x 8488742

[mpr1881-bib-0030] Rothman, K. J., Greenland, S., & Lash, T. L. (2008). Modern epidemiology. Lippincott Williams & Wilkins.

[mpr1881-bib-0031] Rugkåsa, J. (2016). Effectiveness of community treatment orders: The international evidence. Canadian Journal of Psychiatry, 61(1), 15–24. 10.1177/0706743715620415 27582449 PMC4756604

[mpr1881-bib-0032] Salize, H. J., & Dressing, H. (2004). Epidemiology of involuntary placement of mentally ill people across the European Union. British Journal of Psychiatry, 184(2), 163–168. 10.1192/bjp.184.2.163 14754830

[mpr1881-bib-0033] Saya, A., Brugnoli, C., Piazzi, G., Liberato, D., Di Ciaccia, G., Niolu, C., & Siracusano, A. (2019). Criteria, procedures, and future prospects of involuntary treatment in psychiatry around the world: A narrative review. Frontiers in Psychiatry, 10, 271. 10.3389/fpsyt.2019.00271 31110481 PMC6501697

[mpr1881-bib-0034] Schloerke, B., Cook, D., Larmarange, J., Briatte, F., Marbach, M., Thoen, E., Elberg, A., Toomet, O., Crowley, J., Hofmann, H., & Wickham, H. (2020). GGally: Extension to ‘ggplot2’ (2.0.0) [Computer software]. https://CRAN.R‐project.org/package=GGally

[mpr1881-bib-0035] Sheridan Rains, L., Zenina, T., Dias, M. C., Jones, R., Jeffreys, S., Branthonne‐Foster, S., Lloyd‐Evans, B., & Johnson, S. (2019). Variations in patterns of involuntary hospitalisation and in legal frameworks: An international comparative study. The Lancet Psychiatry, 6(5), 403–417. 10.1016/S2215-0366(19)30090-2 30954479 PMC6475657

[mpr1881-bib-0036] Statistics Norway . (2020). 07459: Population, by sex and one‐year age groups (M) 1986—2020. PX‐Web SSB. http://www.ssb.no/en/statbanken/statbank/table/07459/

[mpr1881-bib-0037] Weich, S., McBride, O., Twigg, L., Duncan, C., Keown, P., Crepaz‐Keay, D., Cyhlarova, E., Parsons, H., Scott, J., & Bhui, K. (2017). Variation in compulsory psychiatric inpatient admission in England: A cross‐classified, multilevel analysis. The Lancet Psychiatry, 4(8), 619–626. 10.1016/S2215-0366(17)30207-9 28647537

[mpr1881-bib-0038] Wennberg, J. E. (2010). Tracking medicine: A researcher's quest to understand health care. Oxford University Press.

[mpr1881-bib-0040] White, R. (2019). fhidata: Structural data for Norway (2019.8.27). [Computer software]. https://CRAN.R‐project.org/package=fhidata

[mpr1881-bib-0041] Wickham, H., Averick, M., Bryan, J., Chang, W., McGowan, L., François, R., Grolemund, G., Hayes, A., Henry, L., Hester, J., Kuhn, M., Pedersen, T., Miller, E., Bache, S., Müller, K., Ooms, J., Robinson, D., Seidel, D., Spinu, V., …, Yutani, H. (2019). Welcome to the tidyverse. Journal of Open Source Software, 4(43), 1686.

[mpr1881-bib-0042] Wierdsma, A. I., & Mulder, C. L. (2009). Does mental health service integration affect compulsory admissions? International Journal of Integrated Care, 9, e90. 19777114 10.5334/ijic.324 PMC2748183

[mpr1881-bib-0043] Zinkler, M., & Priebe, S. (2002). Detention of the mentally ill in Europe—A review. Acta Psychiatrica Scandinavica, 106(1), 3–8. 10.1034/j.1600-0447.2002.02268.x 12100342

