# Corrigendum

**DOI:** 10.1002/mpr.1911

**Published:** 2022-06-01

In the article entitled “Measuring the level of compulsory hospitalisation in mental health care: The performance of different measures across areas and over time”, Hofstad et al., 2021 the following first two sentences ‘The number of compelled inpatients is necessarily less than or equal to the number of events in a period. Consequently, measures based on inpatient counts will by necessity never display more variation than measures based on hospitalisation counts when applied to the same dataset.’ under the Section 4.1 have been corrected as below:

The number of compelled inpatients is necessarily less than or equal to the number of events in a period, and the magnitude of geographical variation can differ substantially between the two measures.

We apologize for this error.
