# Supplementary material for: Measuring the level of compulsory hospitalisation in mental health care: The performance of different measures across areas and over time
Source: Int J Methods Psychiatr Res. 2021 May 25;30(3):e1881. doi: 10.1002/mpr.1881 (PMC8412230; doi:10.1002/mpr.1881)
Supplement: Supplementary file 1 — Supplementary Material S1 [file MPR-30-e1881-s002.pdf]

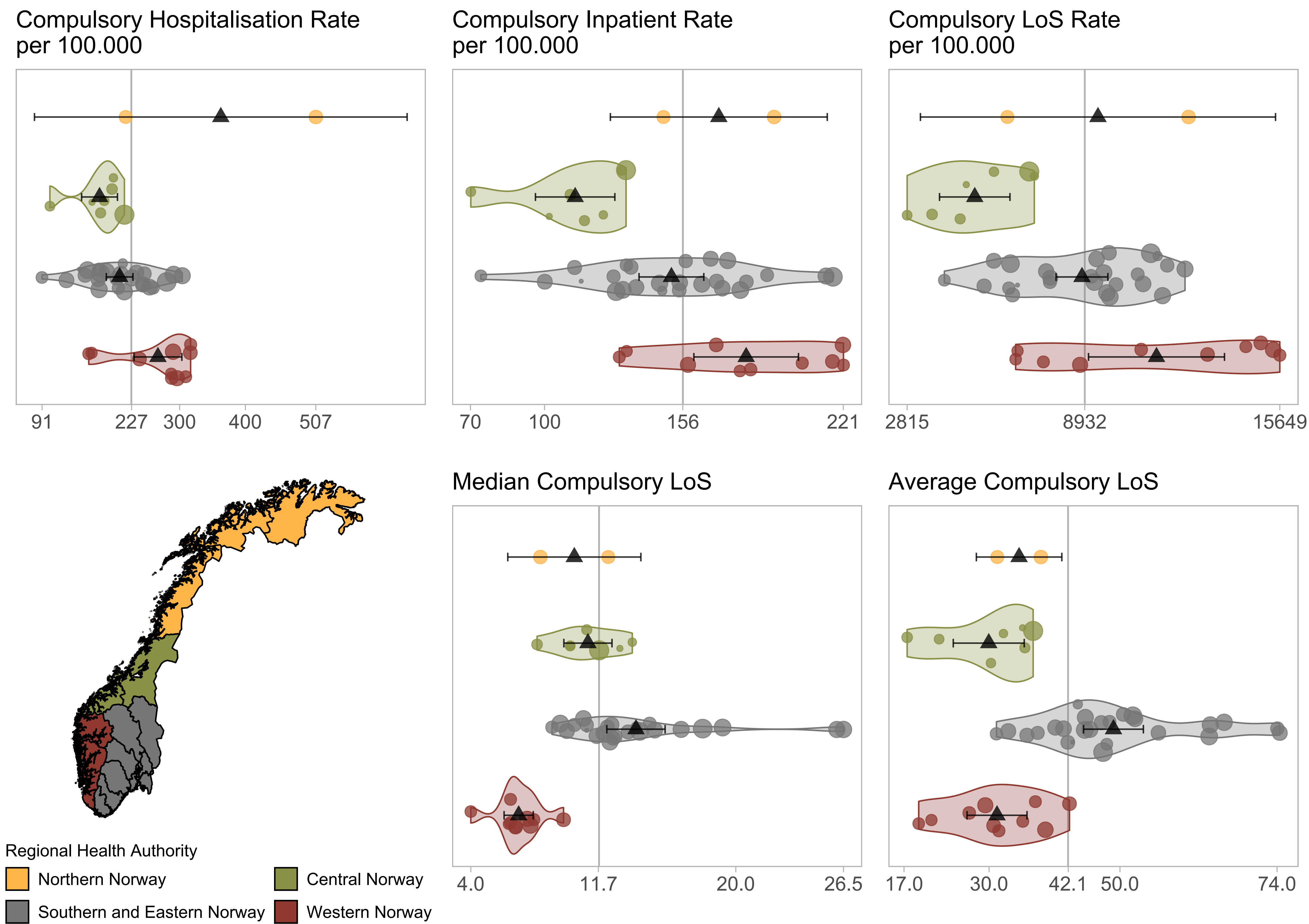
Compulsory Hospitalisation Rate per 100.000
91
227
300
400
507
Compulsory Inpatient Rate per 100.000
70
100
156
221
Compulsory LoS Rate per 100.000
2815
8932
15649
Median Compulsory LoS
4.0
11.7
20.0
26.5
Average Compulsory LoS
17.0
30.0
42.1
50.0
74.0
Regional Health Authority
Northern Norway
Central Norway
Southern and Eastern Norway
Western Norway
