# Supplementary figures and images for: Measuring the level of compulsory hospitalisation in mental health care: The performance of different measures across areas and over time
Source: Int J Methods Psychiatr Res. 2021 May 25;30(3):e1881. doi: 10.1002/mpr.1881 (PMC8412230; doi:10.1002/mpr.1881)

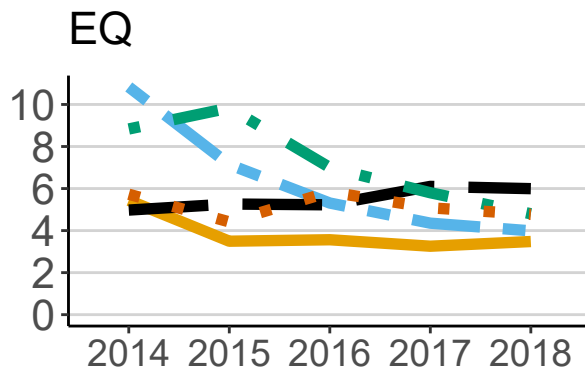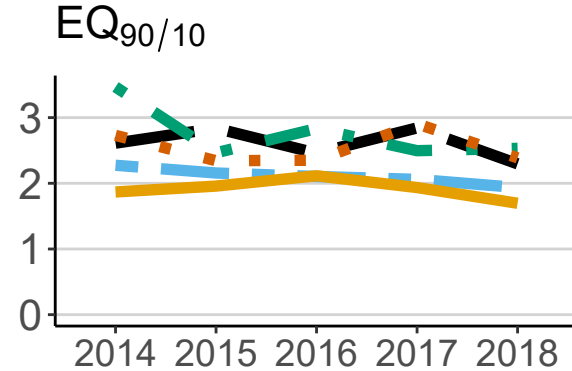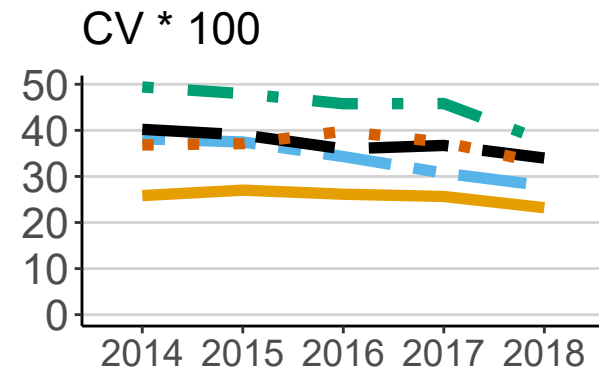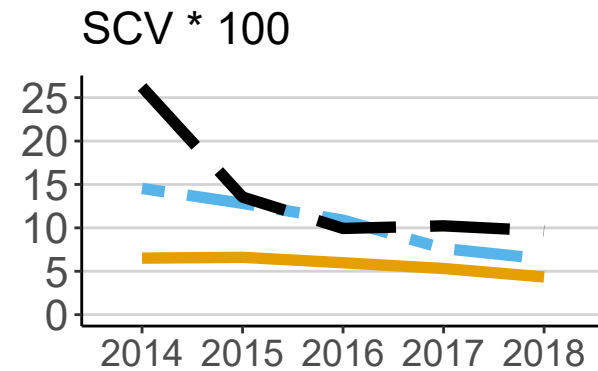

— Hospitalisation rate — Inpatient rate — LoS rate ■ LoS median ■ LoS average

Supplement: Supplementary file 2 — Supplementary Material S2 [file MPR-30-e1881-s004.pdf]

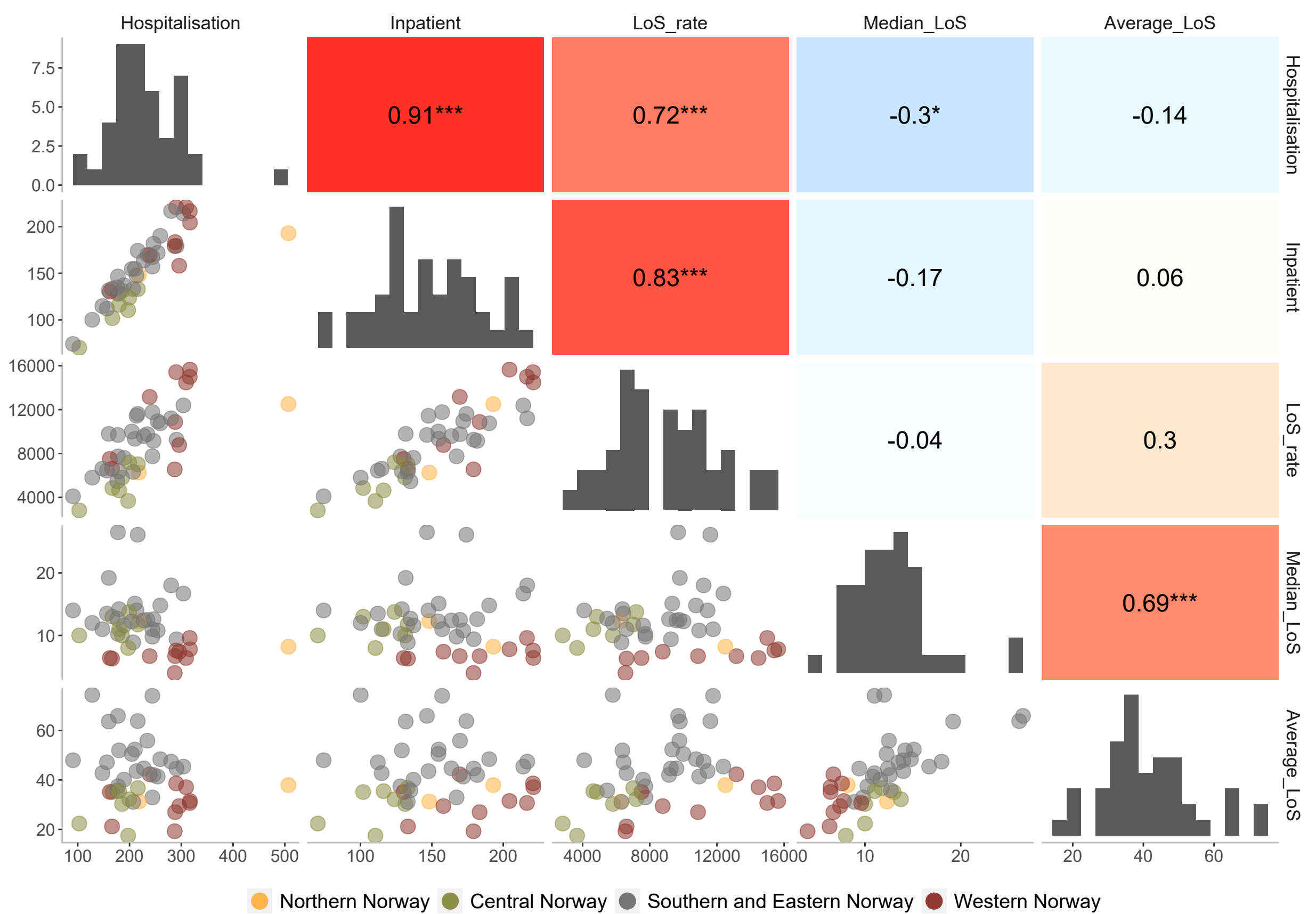

Supplement: Supplementary file 3 — Supplementary Material S3 [file MPR-30-e1881-s001.pdf]

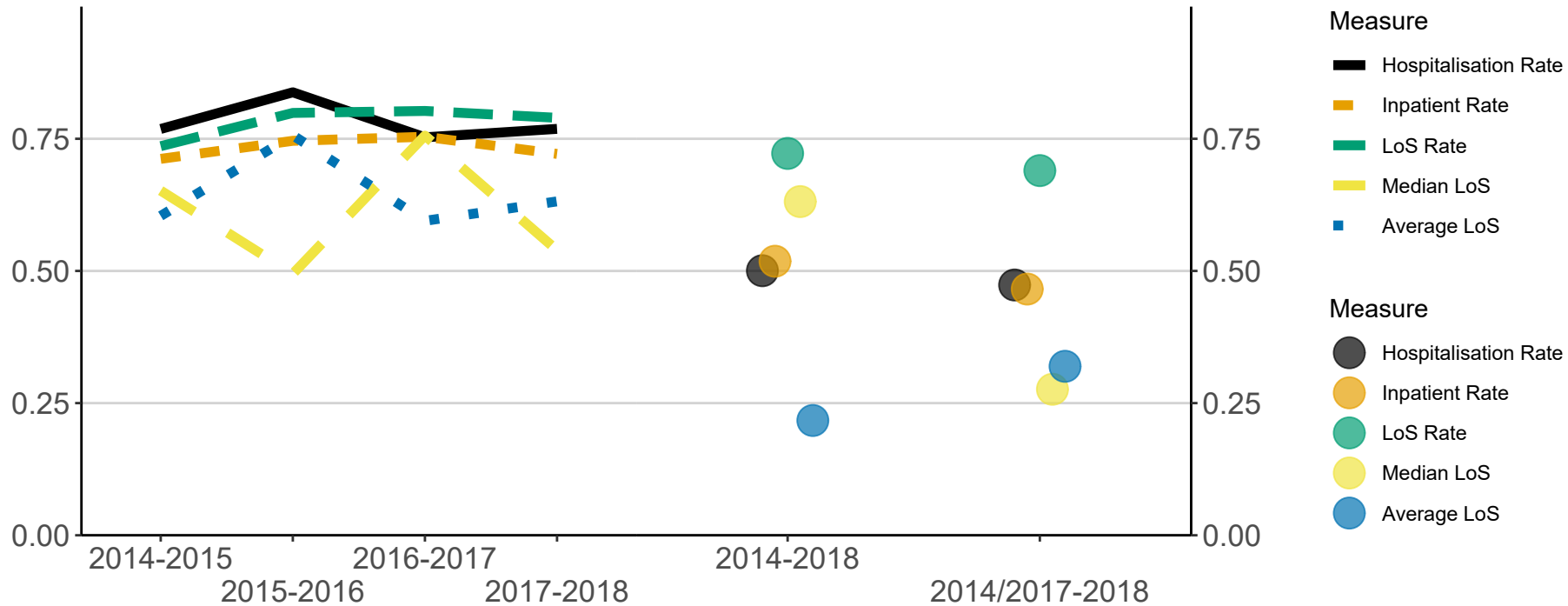

Supplement: Supplementary file 4 — Supplementary Material S4 [file MPR-30-e1881-s003.pdf]
